# Quantitative T2 mapping to characterize the process of intervertebral disc degeneration in a rabbit model

**DOI:** 10.1186/1471-2474-14-357

**Published:** 2013-12-18

**Authors:** Wei Sun, Kai Zhang, Chang-qing Zhao, Wei Ding, Jun-jie Yuan, Qi Sun, Xiao-jiang Sun, You-zhuan Xie, Hua Li, Jie Zhao

**Affiliations:** 1 Shanghai Key Laboratory of Orthopaedic Implant, Department of Orthopaedic Surgery, Shanghai Ninth People’s Hospital, Shanghai Jiao Tong University School of Medicine, 639 Zhizaoju Road, Shanghai 200011, PR China; 2 Department of Radiology, Shanghai Ninth People’s Hospital, Shanghai Jiao Tong University School of Medicine, 639 Zhizaoju Road, Shanghai 200011, PR China

**Keywords:** Intervertebral disc, Degeneration, Animal model, MRI, T2 mapping

## Abstract

**Background:**

To investigate the potential of T2 mapping for characterizing the process of intervertebral disc degeneration (IDD) in a rabbit model.

**Methods:**

Thirty-five rabbits underwent an annular stab to the L4/5 discs (L5/6 discs served as internal normal controls). Degenerative changes were graded according to the modified Thompson classification and quantified in T2 respectively at pre-operation, 1, 3, 6, 12 and 24 weeks postoperatively. After MRI analysis, expression analysis of aggrecan and type II collagen gene in nucleus pulposus (NP) was performed using real time polymerase chain reaction (real-time PCR). The longitudinal changes in NP T2 and gene expressions were studied by repeated measures and ANOVA, linear regression was performed for their correlations through the process of IDD. The reliability analysis of method of measurement of NP T2 was also performed.

**Results:**

There was a strong inverse correlation between NP T2 and Thompson grades (r = -0.85). The decline of L4/5 NP T2 through 24 weeks was nonlinear, the most significant decrease was observed in 3 weeks postoperatively (*P*<0.05). The tendency was confirmed at gene expression levels. NP T2 correlated strongly with aggrecan (R^2^ = 0.85, *P*<0.01) and type II collagen (R^2^ = 0.78, *P*<0.01) gene expressions. The intraclass correlation coefficients for interobserver and intraobserver reliability were 0.963 and 0.977 respectively.

**Conclusions:**

NP T2 correlates well with aggrecan and type II collagen gene expressions. T2 mapping could act as a sensitive, noninvasive tool for quantitatively characterizing the process of IDD in longitudinal study, help better understanding of the pathophysiology of IDD, assist us to detect the degenerative cascade, and develop a T2-based quantification scale for evaluation of IDD and efficacy of therapeutic interventions.

## Background

Degenerative disc disease (DDD) is considered as the most common cause of low back pain, even though the pathophysiological correlation between pain and disc degeneration is not fully understood [1]. Intervertebral disc degeneration (IDD) is characterized by a decrease in proteoglycan content resulting in loss of hydration in the central nucleus pulposus (NP) and collagen degradation, which eventually lead to morphologic changes and alterations in biomechanical properties [2]. T2-weighted magnetic resonance imaging (MRI) is a well-established method for semiquantitative evaluation of IDD, allowing for a grading of IDD by several conventional grading systems with a scale of three to five grades [3] based on morphological changes or changes in the degree and area of signal intensity. T2-weighted MRI is competent to detect late IDD including a loss of T2-weighted MRI signal, disc bulge or herniation and narrowing of disc space, however, it is limited in detecting subtle biochemical changes as representative of early IDD [4]. In addition, this method for evaluation is visual, subjective, and inaccurate because the signal intensity and morphologic changes cannot be measured in absolute terms due to the variable imaging conditions and many arbitrary factors [5]. This may lead to interobserver bias, especially when observers classify relatively minor changes in signal intensity or morphology. These limitations have led to the search for a better diagnostic tool for quantitative evaluation of IDD.

Transverse relaxation time (T2) mapping, a biochemical MRI technique to calculate relaxation time, has the potential to offer a quantitative assessment of IDD [6-8]. T2 is the decay constant for T2 signal intensity in MRI. Unlike T2 signal intensity, T2 is neither scanner nor image parameter dependent [9]. Rather, it reflects an intrinsic property of tissue providing information about water content, collagen orientation and matrix structure [10]. It has been demonstrated that T2 of intervertebral disc (IVD) correlates well with water and proteoglycan content [9]. Thus, quantitative T2 evaluation may be beneficial to detect subtle biochemical changes within IVD that may not be apparent with qualitative or semiquantitative measures.

The efficacy of T2 mapping for quantitative evaluation of IDD has been demonstrated in previous studies, most of which were cross-sectional [11-13]. However, the potential role of T2 mapping in longitudinal study on IDD was not fully investigated, because IDD is a multifaceted chronic process and it is difficult to perform a longitudinal study on patients. The purpose of this study is to investigate the potential applications of T2 mapping in longitudinal study as a noninvasive tool for quantitatively characterizing the process of IDD, by studying the changes in NP T2 and extracellular matrix (ECM)-related gene expressions in the progression of IDD in a rabbit model that allows longitudinal correlation of T2 and biochemical parameter for IDD.

## Methods

### Animal and surgical procedure

Thirty-five healthy female New Zealand white rabbits (aged 1 year, and weighting between 2.5 and 3.0 kg) were used for this study. The experimental protocol was approved by the Animal Care and Experiment Committee of Shanghai Jiaotong University School of Medicine.

Disc degeneration was induced via a validated rabbit puncture method [14]. The rabbits were tranquilized by intramuscular injection of ketamine hydrochloride (40 mg/kg) and xylazine (2.5 mg/kg). Under general anesthesia, the rabbits’ spines were exposed from an anterolateral retroperitoneal approach. L4–L5 discs were punctured with a 16-gauge hypodermic needle to a depth of 5 mm, L5–L6 discs were left undisturbed to serve as the control discs and then the surgical incisions were closed routinely. Postoperatively, the rabbits were housed in individual cages and permitted free activity, food, and water.

### MRI scanning procedures

The study evaluated the L4–L5 and L5–L6 discs of twenty rabbits selected randomly at baseline pre-operation and 1, 3, 6, 12, 24 weeks post-operation for a total of 240 discs. A 3.0 T MR scanner (Magnetom Verio; Siemens Medical Solutions, Erlangen, Germany) and standard human knee coil were used to obtain T2-weighted images (repetition time = 3,800 ms, echo time = 98 ms, 130 × 130 mm field of view, slice thickness was 2 mm with a 0-mm gap ) and T2 mapping images (repetition time = 1,000 ms, echo time = 12.3, 24.6, 36.9, 49.2, and 61.5 ms, 130 × 130 mm field of view, slice thickness was 2 mm with a 0-mm gap). The midsagittal MR image was identified based on spinal cord and spinous processes. Midsagittal T2-weighted images were used for conventional grading, T2 mapping images were used for quantitative measure of NP T2.

### MRI processing

**Figure 1:**
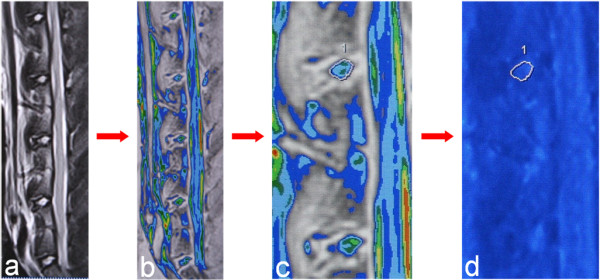
**Illustration of the color-based method for NP T2 measure. (a, b)** To obtain clear indication of NP contour. **(c)** To draw the contour of NP along the largest concentric circle as the region of interest. **(d)** NP T2 of the corresponding region of interest in T2 mapping image can be computed automatically.

Accurate T2 measure requires the clear contour definition of NP. However, it is sometimes challenging to define the clear boundary between NP and endplates of the cranial and caudal vertebral bodies in the sagittal T2-weighted images and T2 mapping images. Here we developed a color-based method to draw the contour of NP. As the first step, we applied color look-up table to the T2-weighted image (Figure 1a) to obtain the clear indication of NP contour (Figure 1b). The second step is to draw the contour of NP along the largest concentric circle as the region of interest (ROI) as depicted in previous study [15] (Figure 1c). Finally, the ROI of the segmented NP was used to identify the location of NP in the corresponding T2 mapping image in the same sagittal plane (Figure 1d), and then NP T2 was computed automatically.

### Image analysis

In the T2-weighted images, the L4/5 and L5/6 discs were classified by two observers (a radiologist with more than 10 years of experience and a special interest in musculoskeletal radiology, and an orthopedic surgeon with 10 years experience), according to the modified Thompson classification from Grade 1 to 4 (1, normal; 2, minimal decrease of signal intensity but obvious narrowing of high signal area; 3, moderate decrease of signal intensity; and 4, severe decrease of signal intensity) depicted in previous study [16]. If the two observers have different opinions in grading a disc, they will turn to the third observer (an orthopedic surgeon with 20 years experience) for giving a final grade to the disc in consensus.

### Inter- and intraobserver analysis

Ten rabbits were selected randomly at each time point for the interobserver evaluation of NP T2 by the aforementioned observers. In addition, the radiologist performed the same analysis twice, with a delay of two months, to assess intraobserver agreement.

### ECM-related gene expressions analysis

After MRI analysis, three rabbits selected randomly were killed at each time point for real time polymerase chain reaction (real-time PCR) analysis. Total ribonucleic acid (RNA) was isolated from NP using Trizol reagent (Invitrogen) according to the manufacturer’s instructions. After reverse transcription reaction, real-time PCR was performed by an ABI 7500 system using SYBR Premix Ex Taq™ (Takara, Dalian, China) according to the manufacturer’s instructions. The conditions of real-time PCR were as follows: 35 cycles at 95°C for 15 s, 60°C for 34 s. Dissociation stage was added to the end of amplification procedure. There was no nonspecific amplification determined by the dissolve curve. Data were normalized to mRNA levels of GAPDH using the △△Ct method to calculate the relative mRNA levels of target gene. The primer sequences used for this analysis were listed in Table 1.

**Table 1:** Sequence of primers used for real-time PCR analysis

**Gene**	**Sequence (5′-3′)**	**Product size (bp)**
Aggrecan	F: TAAACCCGGTGTGAGAACCG	176
	R: CCTGGGTGACAATCCAGTCC	
Collagen II	F: GGATAGACCCCAACCAAGGC	122
	R: GCTGCTCCACCAGTTCTTCT	
GAPDH	F: GGAATCCACTGGCGTCTTCA	122
	R: GGTTCACGCCCATCACAAAC	

### Statistical analysis

Statistical analysis was performed with SPSS 16.0 (SPSS Inc., Chicago, IL, USA). ANOVA and repeated measures were applied for multiple comparisons of different Thompson grades and for pairwise comparisons for different time points. The correlation of NP T2 and the Thompson grading and linear regression of NP T2 versus gene expressions were performed. For inter- and intraobserver reliability analysis, we employed the intraclass correlation coefficient (ICC). The significance level was set at 0.05.

## Results

The T2-weighted image-based modified Thompson classification consisted of the following: grade 1, 146 (60.8%) discs; grade 2, 40 (16.7%) discs; grade 3, 32 (13.3%) discs; and grade 4, 22 (9.2%) discs. The mean NP T2 in milliseconds for different Thompson grades is shown in Table 2.

**Table 2:** T2 for NP with different Thompson grades

**Thompson grade**	**1**	**2**	**3**	**4**	***P*-value**
Number of discs	146	40	32	22	
NP T2 (ms)	121.6 ± 10.4	87.5 ± 16.4	64.2 ± 11.5	45.4 ± 7.9	<0.01

Mean values (±standard deviation) of T2 relaxation time in milliseconds for NP for different Thompson grades are shown. NP: nucleus pulposus.

**Figure 2:**
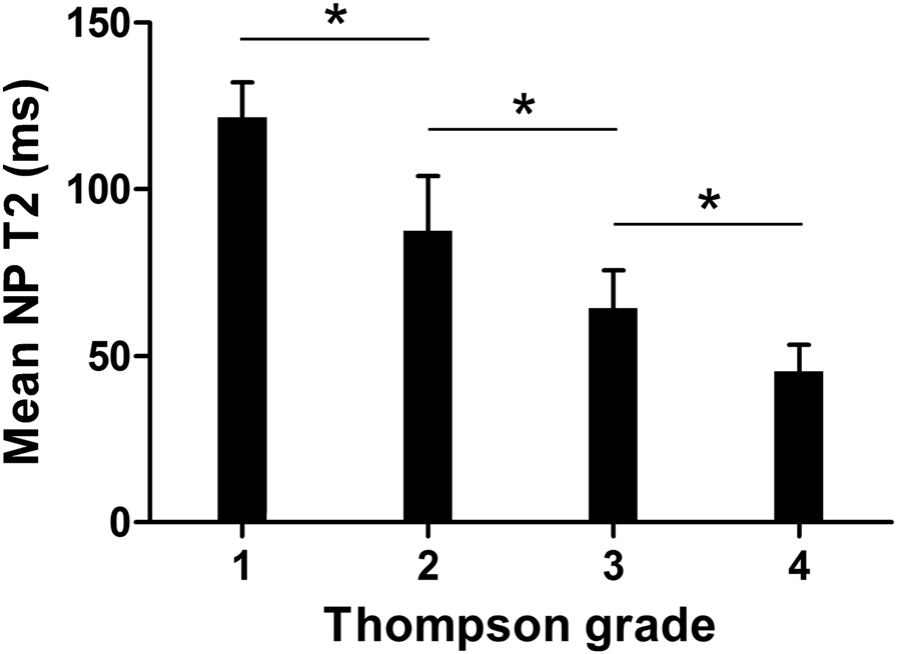
**Mean nucleus pulposus (NP) T2 for different Thompson grades.** A stepwise decrease in NP T2 can be observed from Thompson grade 1 to 4. Error bars represent the standard deviation from the mean. * *P*<0.01.

There was a stepwise decrease in NP T2 with increasing grade, and highly significant differences were observed between the grades (*P*<0.01; Table 2 and Figure 2). The Spearman correlation showed a strong inverse correlation between NP T2 and the Thompson grade (r = -0.85; *P*<0.01; Figure 2).

**Figure 3:**
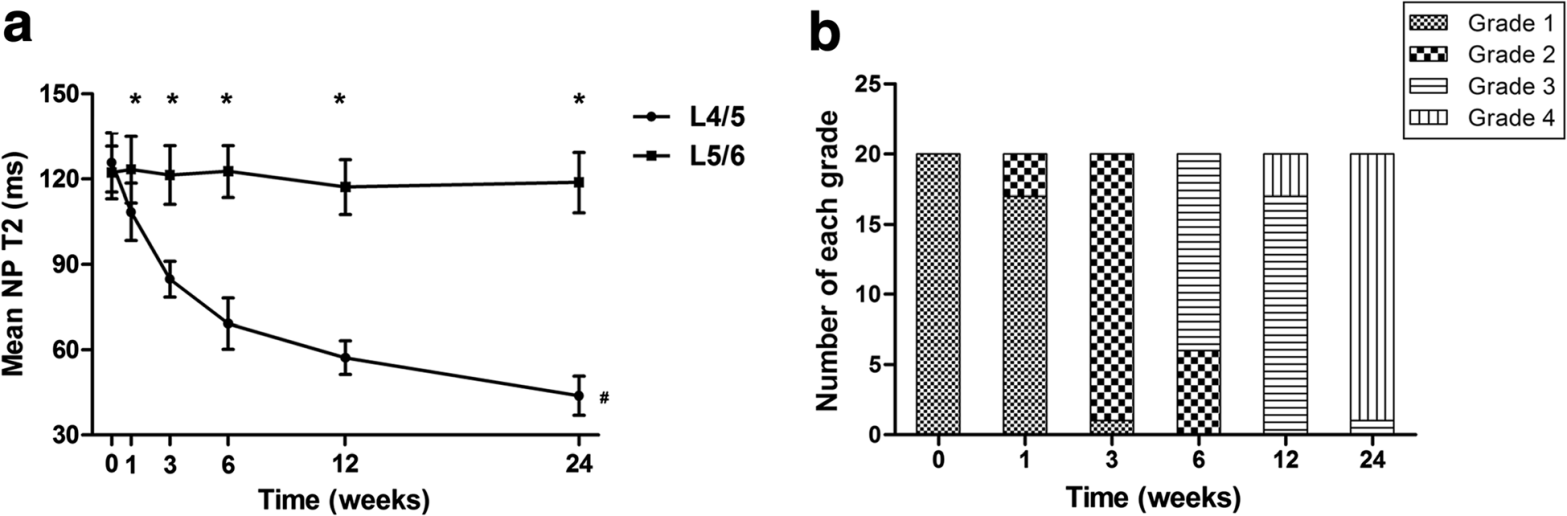
**Mean nucleus pulposus (NP) T2 and Thompson grading at each time point. (a)** A significant decrease in T2 for L4/5 NP can be observed with time (^*#*^*P*<0.05), especially in 3 weeks postoperatively, while no significant decrease in T2 for L5/6 NP over the 24-week period. At 1 week postoperatively, the mean T2 for L4/5 NP was less than that of L5/6 NP and remained so through 24 weeks (*P<0.05). Error bars represent the standard deviation from the mean. **(b)** The Thompson grading for L4/5 discs at each time point.

The L4/5 NP T2 decreased progressively over the 24-week period. The significant decrease in T2 was observed in 3 weeks postoperatively. Then the decline began to slow, especially from 12 weeks to 24 weeks postoperatively (*P*<0.05; Figure 3a). In contrast, no significant decrease in T2 for the L5/6 NP was observed through the 24-week period (*P*>0.05; Figure 3a). At 1 week postoperatively, seventeen out of twenty L4/5 discs were grade 1, while the mean T2 for the L4/5 NP was already significantly less than that of the corresponding control L5/6 NP (*P*<0.05; Figure 3). Most of the L4/5 discs were not classified as grade 2 until 3 week postoperatively (Figure 3b). The mean NP T2 for Thompson grade 2 discs was significantly less than that of discs at 1 week postoperatively (*P*<0.05), while showed no significant difference when compared to that of discs at 3 weeks postoperatively (*P*>0.05).

**Figure 4:**
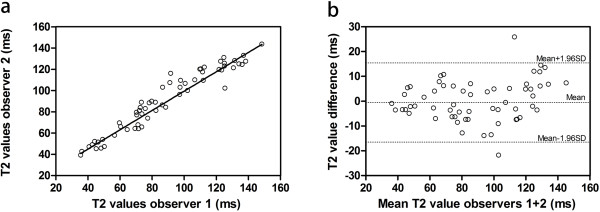
**Scatter-dot plots of interobserver data, including Bland-Altman plot. (a)** The Scatter-dot plot shows the high degree of agreement between interobserver measurements. **(b)** Bland-Altman plot shows the T2 difference between two observers on the y-axis and the mean T2 values of two observers on the x-axis, which indicates a good repeatability.

The interobserver analysis revealed strong agreement between the two observers (maximum deviation = 12.5), and the ICC for interobserver reliability was 0.963 (Table 3, Figure 4). Similar result was obtained for the intraobserver analysis, with an ICC of 0.977, which corresponded to a very strong intraobserver agreement (maximum deviation = 7.8) (Table 3).

**Table 3:** Inter- and Intraobserver reliability analysis

	**Pearson’s correlation**	**ICC**^**a**^	***P*-value**
Interobserver analysis	0.964	0.963	<0.001
Intraobserver analysis	0.977	0.977	<0.001

^a^ICC: intraclass correlation coefficient.

**Figure 5:**
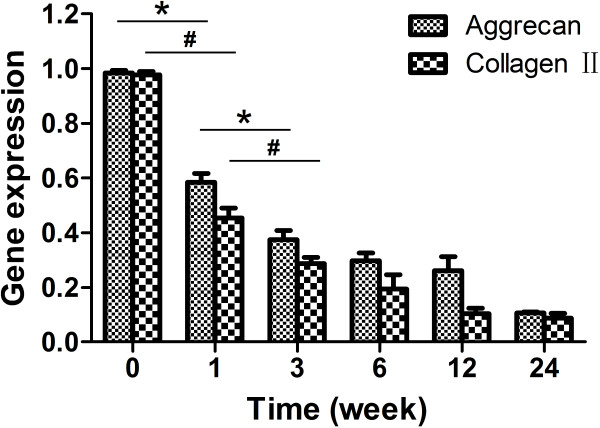
**Relative gene expressions of aggrecan and type II collagen in L4/5 NP at different time points.** Aggrecan and type II collagen mRNA decreased markedly from pre-op to 3 weeks postoperatively, and then declined gradually with time. ^*^P<0.05, ^*#*^*P*<0.05.

Figure 5 illustrates the relative gene expressions of aggrecan and type II collagen in L4/5 NP at different time points. Aggrecan and type II collagen mRNA decreased markedly from pre-op to 3 weeks postoperatively, and then declined gradually with time (*P*<0.05). In contrast, the L5/6 NP relatively maintained its gene expression levels over the 24-week period (*P*>0.05).

**Figure 6:**
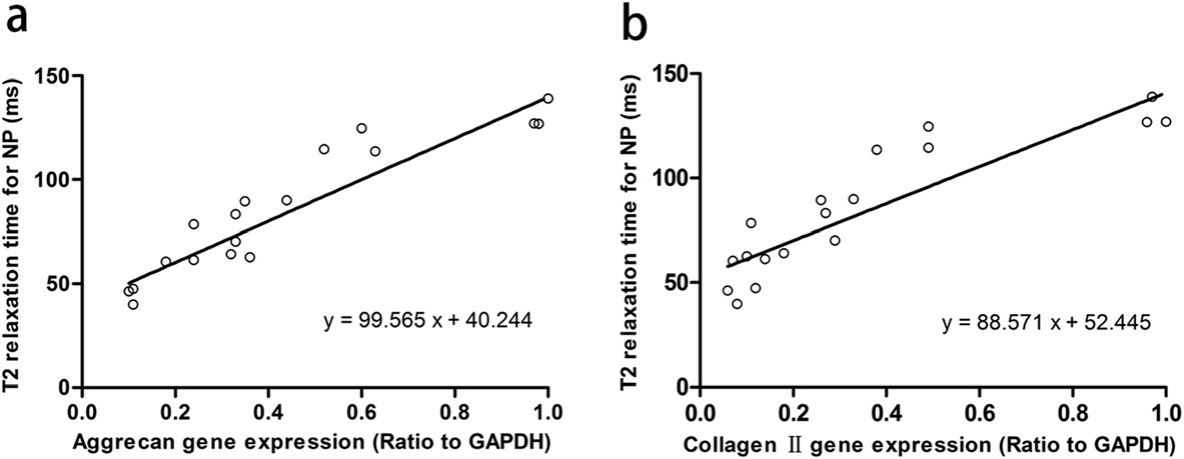
**T2 relaxation time plotted against gene expressions in L4/5 NP. (a)** T2 relaxation time plotted against aggrecan gene expression. R^2^ is 0.85 and *P*<0.01. **(b)** T2 relaxation time plotted against type II collagen gene expression. R^2^ is 0.78 and *P*<0.01.

For NP, T2 and aggrecan gene expression correlated strongly (R^2^ = 0.85, *P*<0.01). The slope of this correlation was 99.565 (Figure 6a). T2 also correlated with type II collagen gene expression (R^2^ = 0.78, *P*<0.01). The slope was 88.571 (Figure 6b).

## Discussion

This study investigated the longitudinal changes in NP T2 and expressions of aggrecan and type II collagen gene as well as their correlations in the progression of IDD in a rabbit model over 24 weeks, further demonstrating the potential applications of T2 mapping in future clinical and experimental research.

NP T2 decreased with increasing Thompson grade, which is consistent with other published studies on human degenerative IVDs [8,12], likely reflecting a decrease in proteoglycan and water content. Benneker et al. [17] and Schiebler et al. [18] suggested that early IDD may exist before there is a loss of signal intensity in T2-weighted image. Our outcomes demonstrated that T2-weighted MRI could not detect degenerative changes in most of the discs until 3 weeks postoperatively, as Sobajima’s report [14], while T2 mapping was able to detect IDD at 1 week postoperatively or earlier. With T2 mapping’s high degree of sensitivity and accuracy, IDD can be detected at an earlier stage, consequently patients may benefit from alternative therapies (e.g., dynamic stabilization [19], biologic therapies [20]) rather than spinal fusion.

T2 mapping could provide a reliable, continuous scale for quantitatively evaluating IVD and characterizing the process of IDD rather than a discrete, ordinal one as assessed by the conventional classifications that fail to identify lesser degree of progression of IDD. T2 mapping has the potential for detecting certain vital events in the process of IDD, such as the intervertebral disc degenerative cascade which refers to nonreversible cell-mediated responses leading to further disruption and is known to encompass disruption of ECM [20,21]. The development of noninvasive imaging method may revolutionize our approaches to understanding IDD. The longitudinal change in NP T2 suggested the process of IDD was nonlinear, and the significant progression occurred in 3 weeks postoperatively. The tendency of IDD and the marked progression were also confirmed at the gene expression levels. Similarly, Sobajima et al. reported the aggrecan and collagen type II mRNA levels had decreased markedly by 3 weeks postoperatively [22]. Accordingly, we may presume to some extent that the accelerated degeneration of this rabbit model occurred within 3 weeks postoperatively. To our knowledge, this study may be the first to investigate the accelerated degeneration by monitoring the longitudinal changes in NP T2 and expressions of ECM-related gene through the process of IDD. NP T2 correlated well with aggrecan gene expression, confirming the established relationship between T2 and proteoglycan at gene expression level. In addition, the results showed that decreased NP T2 was associated well with down-regulated type II collagen gene expression, likely reflecting the disorganization and degradation of the collagen network in the process of IDD [10,23]. Accordingly, T2 might also have potential in the visualization of collagen integrity in the disc. Our data further supported that T2 mapping may act as a noninvasive tool for characterizing the process of IDD in a continuous quantitative manner rather than a discrete, ordinal one, particularly in longitudinal studies. This will help our better understanding of the complex pathophysiology of human IDD and detection of certain biologic processes in the course of IDD such as the degenerative cascade. Furthermore, this is of importance because it has been presumed that there may be a time point beyond which disc degeneration will become irreversible [24,25], T2 mapping may provide evidence for us to make sure therapeutic interventions are taken at the early stage before the onset of the degenerative cascade which might no longer be retarded once initiated [21]. This will be very valuable in retarding or reversing IDD.

Prior in vivo experimental studies have drawn an encouraging conclusion that emerging biologic therapies including growth factor therapy [26], gene therapy [27], cell therapy [28], and tissue engineering approaches [29] have a beneficial efficacy in slowing IDD. However, the timing for application of therapeutic interventions varied from 2 weeks to 4 weeks after IDD was induced [27,28,30], what degree of IDD can be repaired or regenerated remains unclear. The reason is that there is not a standardized quantification scale for evaluation of IDD. Takashima et al. [31] have proposed that the T2 value-based grade scale in their study may be useful for future research on IDD, with a high degree of objectivity. Watanabe et al. [5] and Perry et al. [7] also suggested that T2 mapping may provide a T2-based classification and the new system may be able to detect early degenerative changes before the conventional classification systems can. Chan et al. [32] and Malonzo et al. [33] also demonstrated in a papain-induced in vitro disc degeneration model that different severities of biochemical changes (proteoglycan and water loss, and collagen disorganization) could be well reflected by T2. The conventional ranked scales based on T2-weighted MRI are limited in identifying lesser degrees of progression or regression of degeneration in the disc. To evaluate emerging novel therapies for treatment of IDD such as gene therapy a more sensitive tool is required. T2 could be potentially used to develop a reliable continuous scale with higher sensitivity for tracking lesser degrees of progression or regression of IDD, in particular for the assessment of disc regenerative strategies that aim to halt or reverse IDD. With this objective unified scale, studies from different research centers may be correlated with each other and put together for analysis.

The MR image processing method used in this study facilitates us to obtain the accurate NP T2. With a high degree of reliability and repeatability, our method deserved recommended in future clinical and experimental studies. Though some studies [8,12] recommended measuring five areas of IVD divided equally to decrease variance, the middle three areas include the regions of low signal intensity which may compromise the accuracy of NP T2 measurement.

Several limitations are pertinent to this study. First, this animal model provided only an acute model of IDD, which may not truly represent the process of human disc degeneration. Second, other than in natural disc degeneration, the remaining disc cell viability was not affected in this annular stab-induced model. However, it is not likely to affect us to determine T2 mapping’s potential for quantitatively evaluating IVD and characterizing the process of IDD. Third, the small number of specimens and ECM-related gene limited the power of the regression analysis to demonstrate the potential of T2 mapping for characterizing the process of IDD. Furthermore, saggital T2 mapping might need to be combined with axial T2 mapping [5,11] to calculate T2 representing the entire NP in future studies.

## Conclusions

NP T2 correlated well with aggrecan and type II collagen gene expressions. T2 mapping proved to be a sensitive, noninvasive tool for quantitatively characterizing the process of IDD in longitudinal study. It could play an important role in our better understanding of the pathophysiology of IDD, assist us to detect certain biologic processes in the course of IDD such as the degenerative cascade, and develop a T2-based quantification scale for evaluation of IDD and efficacy of therapeutic interventions. This study provides promising new evidence to justify further application of T2 mapping in future clinical and experimental studies on IDD, particularly for longitudinal studies.

## Abbreviations

DDD: Degenerative disc disease; IDD: Intervertebral disc degeneration; NP: Nucleus pulposus; PCR: Polymerase chain reaction; IVD: Intervertebral disc; ECM: Extracellular matrix; ROI: Region of interest; RNA: Ribonucleic acid; AF: Annulus fibrosus; ICC: Intraclass correlation coefficients.

## Competing interests

The authors declare that they have no competing interests.

## Authors’ contributions

WS, KZ and JJY performed animal experiments. CQZ and WD carried out PCR analysis. JZ and WS conceived of the study and participated in its design. WS drafted the manuscript. HL and QS performed MRI evaluation.YZX and XJS performed the statistical analysis. All authors read and approved the final manuscript.

## Pre-publication history

The pre-publication history for this paper can be accessed here:

http://www.biomedcentral.com/1471-2474/14/357/prepub

## References

[B1] Luoma K Riihimaki H Luukkonen R Raininko R Viikari-Juntura E Lamminen A Low back pain in relation to lumbar disc degeneration Spine (Phila Pa 1976) 2000 14 4 487 492 10.1097/00007632-200002150-00016 10707396

[B2] Buckwalter JA Aging and degeneration of the human intervertebral disc Spine (Phila Pa 1976) 1995 14 11 1307 1314 7660243 10.1097/00007632-199506000-00022

[B3] Kettler A Wilke HJ Review of existing grading systems for cervical or lumbar disc and facet joint degeneration Eur Spine J 2006 14 6 705 718 10.1007/s00586-005-0954-y 16172902 PMC3489462

[B4] Wang C Auerbach JD Witschey WR Balderston RA Reddy R Borthakur A Advances in magnetic resonance imaging for the assessment of degenerative disc disease of the lumbar spine Semin Spine Surg 2007 14 2 65 71 10.1053/j.semss.2007.04.009 18037984 PMC2084362

[B5] Watanabe A Benneker LM Boesch C Watanabe T Obata T Anderson SE Classification of intervertebral disk degeneration with axial T2 mapping AJR Am J Roentgenol 2007 14 4 936 942 10.2214/AJR.07.2142 17885068

[B6] Marinelli NL Haughton VM Anderson PA T2 relaxation times correlated with stage of lumbar intervertebral disk degeneration and patient age AJNR Am J Neuroradiol 2010 14 7 1278 1282 10.3174/ajnr.A2080 20360340 PMC7965459

[B7] Perry J Haughton V Anderson PA Wu Y Fine J Mistretta C The value of T2 relaxation times to characterize lumbar intervertebral disks: preliminary results AJNR Am J Neuroradiol 2006 14 2 337 342 16484406 PMC8148766

[B8] Takashima H Takebayashi T Yoshimoto M Terashima Y Tsuda H Ida K Yamashita T Correlation between T2 relaxation time and intervertebral disk degeneration Skeletal Radiol 2011 14 2 163 167 21424906 10.1007/s00256-011-1144-0

[B9] Marinelli NL Haughton VM Munoz A Anderson PA T2 relaxation times of intervertebral disc tissue correlated with water content and proteoglycan content Spine (Phila Pa 1976) 2009 14 5 520 524 10.1097/BRS.0b013e318195dd44 19247172

[B10] Link TM Stahl R Woertler K Cartilage imaging: motivation, techniques, current and future significance Eur Radiol 2007 14 5 1135 1146 10.1007/s00330-006-0453-5 17093967

[B11] Hoppe S Quirbach S Mamisch TC Krause FG Werlen S Benneker LM Axial T2 mapping in intervertebral disc: a new technique for assessment of intervertebral disc degeneration Eur Radiol 2012 14 9 2013 2019 10.1007/s00330-012-2448-8 22544293

[B12] Stelzeneder D Welsch GH Kovács BK Goed S Paternostro-Sluga T Vlychou M Friedrich K Mamisch TC Trattnig S Quantitative T2 evaluation at 3.0T compared to morphological grading of the lumbar intervertebral disc: a standardized evaluation approach in patients with low back pain Eur J Radiol 2012 14 2 324 330 10.1016/j.ejrad.2010.12.093 21315527

[B13] Trattnig S Stelzeneder D Goed S Reissegger M Mamisch TC Paternostro-Sluga T Weber M Szomolanyi P Welsch GH Lumbar intervertebral disc abnormalities: comparison of quantitative T2 mapping with conventional MR at 3.0 T Eur Radiol 2010 14 11 2715 2722 10.1007/s00330-010-1843-2 20559835

[B14] Sobajima S Kompel JF Kim JS Wallach CJ Robertson DD Vogt MT Kang JD Gilbertson LG A slowly progressive and reproducible animal model of intervertebral disc degeneration characterized by MRI, X-ray, and histology Spine (Phila Pa 1976) 2005 14 1 15 24 15626975 10.1097/01.brs.0000148048.15348.9b

[B15] Bechara BP Leckie SK Bowman BW Davies CE Woods BI Kanal E Sowa GA Kang JD Application of a semiautomated contour segmentation tool to identify the intervertebral nucleus pulposus in MR images AJNR Am J Neuroradiol 2010 14 9 1640 1644 10.3174/ajnr.A2162 20581067 PMC7964996

[B16] Masuda K Aota Y Muehleman C Imai Y Okuma M Thonar EJ Andersson GB An HS A novel rabbit model of mild, reproducible disc degeneration by an anulus needle puncture: correlation between the degree of disc injury and radiological and histological appearances of disc degeneration Spine (Phila Pa 1976) 2005 14 1 5 14 15626974 10.1097/01.brs.0000148152.04401.20

[B17] Benneker LM Heini PF Anderson SE Alini M Ito K Correlation of radiographic and MRI parameters to morphological and biochemical assessment of intervertebral disc degeneration Eur Spine J 2004 14 1 27 35 15723249 10.1007/s00586-004-0759-4 PMC3476685

[B18] Schiebler ML Camerino VJ Fallon MD Zlatkin MB Grenier N Kressel HY In vivo and ex vivo magnetic resonance imaging evaluation of early disc degeneration with histopathologic correlation Spine 1991 14 6 635 10.1097/00007632-199106000-00007 1862402

[B19] Cho BY Murovic J Park KW Park J Lumbar disc rehydration postimplantation of a posterior dynamic stabilization system J Neurosurg Spine 2010 14 5 576 580 10.3171/2010.5.SPINE08418 21039146

[B20] Kepler CK Anderson DG Tannoury C Ponnappan RK Intervertebral disk degeneration and emerging biologic treatments J Am Acad Orthop Surg 2011 14 9 543 553 21885700 10.5435/00124635-201109000-00005

[B21] Adams MA Roughley PJ What is intervertebral disc degeneration, and what causes it? Spine (Phila Pa 1976) 2006 14 18 2151 2161 10.1097/01.brs.0000231761.73859.2c 16915105

[B22] Sobajima S Shimer AL Chadderdon RC Kompel JF Kim JS Gilbertson LG Kang JD Quantitative analysis of gene expression in a rabbit model of intervertebral disc degeneration by real-time polymerase chain reaction Spine J 2005 14 1 14 23 10.1016/j.spinee.2004.05.251 15653081

[B23] Burstein D Gray ML Is MRI fulfilling its promise for molecular imaging of cartilage in arthritis? Osteoarthritis Cartilage 2006 14 11 1087 1090 10.1016/j.joca.2006.07.001 16901724

[B24] Kozaci LD Guner A Oktay G Guner G Alterations in biochemical components of extracellular matrix in intervertebral disc herniation: role of MMP-2 and TIMP-2 in type II collagen loss Cell Biochem Funct 2006 14 5 431 436 10.1002/cbf.1250 16142692

[B25] Zhao CQ Wang LM Jiang LS Dai LY The cell biology of intervertebral disc aging and degeneration Ageing Res Rev 2007 14 3 247 261 10.1016/j.arr.2007.08.001 17870673

[B26] An HS Takegami K Kamada H Nguyen CM Thonar EJM Singh K Andersson GB Masuda K Intradiscal administration of osteogenic protein-1 increases intervertebral disc height and proteoglycan content in the nucleus pulposus in normal adolescent rabbits Spine 2005 14 1 25 15626976 10.1097/01.brs.0000148002.68656.4d

[B27] Leckie SK Bechara BP Hartman RA Sowa GA Woods BI Coelho JP Witt WT Dong QD Bowman BW Bell KM Injection of AAV2-BMP2 and AAV2-TIMP1 into the nucleus pulposus slows the course of intervertebral disc degeneration in an in vivo rabbit model Spine J 2012 14 1 7 20 10.1016/j.spinee.2011.09.011 22023960 PMC4896143

[B28] Yang F Leung VYL Luk KDK Chan D Cheung KMC Mesenchymal stem cells arrest intervertebral disc degeneration through chondrocytic differentiation and stimulation of endogenous cells Mol Ther 2009 14 11 1959 1966 10.1038/mt.2009.146 19584814 PMC2835041

[B29] Leung V Tam V Chan D Chan BP Cheung K Tissue engineering for intervertebral disk degeneration Orthop Clin North Am 2011 14 4 575 10.1016/j.ocl.2011.07.003 21944593

[B30] Hiyama A Mochida J Iwashina T Omi H Watanabe T Serigano K Tamura F Sakai D Transplantation of mesenchymal stem cells in a canine disc degeneration model J Orthop Res 2008 14 5 589 600 10.1002/jor.20584 18203202

[B31] Takashima H Takebayashi T Yoshimoto M Terashima Y Tsuda H Ida K Yamashita T Correlation between T2 relaxation time and intervertebral disk degeneration Skeletal Radiol 2012 14 2 163 167 10.1007/s00256-011-1144-0 21424906

[B32] Chan SC Bürki A Bonél HM Benneker LM Gantenbein-Ritter B Papain-induced in vitro disc degeneration model for the study of injectable nucleus pulposus therapy Spine J 2013 14 3 273 283 10.1016/j.spinee.2012.12.007 23353003

[B33] Malonzo C Chan SC Kabiri A Eglin D Grad S Bonél HM Benneker LM Gantenbein-Ritter B **A papain-induced disc degeneration model for the assessment of thermo-reversible hydrogel-cells therapeutic approach** J Tissue Eng Regen Med 2013 doi: 10.1002/term.1667. [Epub ahead of print] 10.1002/term.1667 23303720

